# Impact of the Japanese Government's ‘General Principles of Suicide Prevention Policy’ on youth suicide from 2007 to 2022

**DOI:** 10.1192/bjo.2023.616

**Published:** 2023-12-19

**Authors:** Ryusuke Matsumoto, Eishi Motomura, Takashi Shiroyama, Motohiro Okada

**Affiliations:** Department of Neuropsychiatry, Division of Neuroscience, Graduate School of Medicine, Mie University, Japan

**Keywords:** Suicide, community mental health teams, epidemiology, psychiatry and law, mental health services

## Abstract

**Background:**

The Japanese Government programme ‘General Principles of Suicide Prevention Policy' (GPSPP) contributed to decreasing suicide mortality rates (SMRs) before the COVID-19 pandemic, but they increased after the pandemic.

**Aims:**

To identify risk factors for youth suicide and the impact of GPSPP on youth suicide.

**Method:**

Annual suicide numbers during 2007–2022 were obtained from government databases. SMRs of student and non-student youths were analysed with a linear mixed-effects model. Interrupted time-series analysis was conducted to investigate temporal relations between three GPSPP periods and SMRs with 52 suicide motives among high school, special vocational school and university students. Multiple regression analysis was conducted to investigate the influence of grade repetition on university student SMRs.

**Results:**

Non-student youth SMRs were higher than student SMRs. School-related (worrying about the future/underachievement), health-related (mainly mental illness) and family-related (conflict with parent and severe verbal reprimands) motives were major motives for student SMRs. During the first GPSPP period (2007–2012), no student SMRs decreased. During the second period (2012–2017), university and special vocational school student SMRs increased, but high school student SMRs were unchanged. In contrast, during the third period (2017–2022), with the exception of male special vocational school students, all SMRs increased. Unexpectedly, long-term grade repetition was negatively associated with health-related SMRs.

**Conclusions:**

These findings suggest that GPSPP-supported programmes in schools partially contributed to student suicide prevention. To suppress increasing student SMRs, social/life support specialists should participate in in-school support services to bolster the social standing and lives of students who repeat grades or experience setbacks.

Suicide is the fourth leading cause of death among 15- to 29-year-olds worldwide and is even more serious among youth in Japan, where it remained the leading cause of death among youth between 2009 and 2021.^1,2^ The youth age group, which is defined as 15–24 years of age by the United Nations and World Health Organization (WHO),^3^ is composed of students (high school, university and special vocational school students) and non-student young adults.^4^ Over the past decade (2009–2019), Japan has successfully reduced suicide mortality rates (SMRs) per 100 000 by approximately 30%, but during the COVID-19 pandemic, suicides in Japan have continuously increased.^5–8^ Individuals under 30 years of age have been identified as being at high risk for increased SMRs during the pandemic;^6,8^ however, recent studies reported that the SMRs of these high-risk groups had already been increasing before the pandemic (in the late 2010s),^5–8^ suggesting that the increasing SMR trends in Japan since 2020 might involve not only pandemic-associated factors, but also other factors that were present well before the pandemic.

## General Principles of Suicide Prevention Policy

The governmental comprehensive suicide prevention programmes ‘General Principles of Suicide Prevention Policy' (GPSPP) and ‘Emergency Fund to Enhance Community-based Suicide Countermeasures’ have been evaluated to play important roles in decreasing SMRs during 2009–2019.^2,9–13^ GPSPP has established guidelines for suicide prevention to be promoted by the government, and has been revised approximately every 5 years (first term: 2007–2012; second term: 2012–2017; third term: 2017–2022) according to the Plan-Do-Check-Act cycle.^9^ The detailed priority major categories of GPSPP in the three periods are described in Supplementary Table 1 available at https://doi.org/10.1192/bjo.2023.616. From the third to fourth years of each period, the Ministry of Health, Labour and Welfare (MHLW) presented a revised draft of the next GPSPP to prefectural governments, and in the fourth to fifth years, the revised draft of each prefecture according to the draft of MHLW was resubmitted to the MHLW. The next governmental GPSPP was finally approved by the cabinet within the fifth year in the previous GPSPP period. Comprehensive government suicide prevention programmes have decreased the SMRs of working-age and elderly generations by enhancing regional welfare/social safety nets and improving regional social protection vulnerabilities across communities, workplaces and schools;^10–13^ however, despite these efforts, the SMR of individuals under 20 years of age did not decrease during 2009–2019.^6,8^ With regard to possible explanations as to why youth suicide did not decrease, it was proposed that the suicide motives of youth differed from other age groups and were insensitive to effective prevention programmes for adults.^14^ To respond to increasing youth suicide, GPSPP was revised to add the following priorities: ‘development of mental health support service in schools’ in the first term, ‘enhancement of support and counselling systems for bullied children and victims of child abuse or sex crimes’ in the second term, and ‘education on how high-risk youth can request support’ and ‘development of suicide prevention programme for children/adolescents’ in the third term.^2^ The ‘2022 White Paper on Suicide Prevention’ published by the MHLW reported that more than 25% of adolescents/youths who suffered from depressive mood or suicidal ideation could not seek nearby support by themselves.^2^ As described above, GPSPP seems to have been reasonably improved by the Plan-Do-Check-Act cycle. Thus, clarifying the background of the increasing number of youth suicides is essential to the development of effective suicide prevention programmes to address the increasing number of youth suicides. Therefore, to elucidate the impact of GPSPP in schools on the SMRs of youth, the fluctuations in the SMRs of high school, university and special vocational school students disaggregated by 52 suicide motives during the first, second and third terms of GPSPP were assessed.

## Method

### Data source

There are two government suicide statistics databases in Japan: Vital Statistics Registration (VSR), which is managed by the MHLW, and Suicide Statistics (SSNPA), which is managed by the National Police Agency (NPA). In Japan, only medical doctors can prepare death certificates, and the Medical Practitioners Law stipulates that abnormal death must be reported to the NPA within 24 h. The NPA must conduct physiological examinations to determine the course of death in all cases with an abnormal cause of death.^2,15^ The SSNPA provides the number of suicides in each region under the jurisdiction of local police stations. The police investigate the personal characteristics and background factors of each suicide case. Since it is impossible to collect suicide motives from the victims themselves, to eliminate subjectivity as much as possible, the police investigate suicide motives based on evidence, including suicide notes, official documentation (e.g. medical certificates and clinical recordings) and testimony from the victim's family. The results of this investigation suggest the different motives for suicide, and these motives are compared against previously compiled lists of motives. In the SSNPA, suicides are classified into seven major categories: health-related, family-related, economic-related, romance-related, employment-related, school-related and other (including 52 subcategories). Detailed explanations of the suicide motives have been described in previous reports,^15–17^ and detailed lists of categories and numbers of suicides disaggregated by motive are described in Supplementary Tables 2–4. The SSNPA published the annual suicide numbers (disaggregated by 52 suicide motives) of high school (15–18 years old), university (approximately 18–22 years old) and special vocational school (individuals who graduated from middle school, high school or university to acquire special qualifications) students during 2007–2022, but only published the annual number of suicides disaggregated by age, with age classified into two categories: <20 years and 20–29 years.

VSR is a cause-of-death statistics database that publishes suicide, homicide, accidental and unexplained death. The VSR published the annual number of suicide disaggregated by age in 5-year intervals (15–19 years and 20–24 years), until 2019. The present study analysed the annual number of suicides in youths of 15–24 years of age. The number of suicides among non-student youth (15–24 years) was calculated by subtracting the suicide numbers among high school, university and special vocational school students from the total number of suicides in the same age group. The numbers of high school, special vocational school, university and grade-repeating university students were obtained from the School Basic Survey of the Ministry of Education, Culture, Sports, Science and Technology (MEXT) (https://www.e-stat.go.jp/en/statistics/00400001).

### Data analysis

SMRs were calculated by dividing the annual suicide numbers disaggregated by sex (male/female), age (15–24 years), school type (high school, special vocational school and university) and non-student youth (subtracting the numbers of high school, special vocational school and university students from the total suicides of 15–24-year-olds) by the population of the corresponding groups in the same year.

To determine the specific fluctuation patterns among students and non-student youth, including trends and the joinpoints of SMRs during 2007–2019, SMRs of total students (high school, special vocational school and university), total youth (15–24 years) and non-student youth were analysed by a joinpoint regression analysis (JPRA) with Joinpoint Regression Program version 4.9.1.0 for Windows (National Cancer Institute, Bethesda, Maryland, USA; see https://surveillance.cancer.gov/joinpoint/download), and a linear mixed-effects model with SPSS for Windows version 28 (IBM, Armonk, New York, USA).^5–7^ To determine the temporal relationship of the GPSPP period and fluctuation patterns (including trends and discontinuity) and their effect size among SMRs during 2007–2022, an interrupted time-series analysis (ITSA) with robust s.e. were conducted with Stata version 17 for Windows (StataCorp, College Station, Texas, USA).^18^ The intervention periods in ITSA were set at 2012 and 2017, since the first-, second- and third-term GPSPP were implemented during 2007–2012, 2012–2017 and 2017–2022, respectively. Furthermore, intervention periods were also set at 2012, 2017 and 2020, to analyse the effects of the COVID-19 pandemic on SMRs. The detailed Stata programs for ITSA were described in a previous report.^18^

The rates of repeating the same grade for 1, 2, 3 and 4 more years were also calculated by dividing the annual number of university students in the same year. To quantify the impact of grade repetition rates on the SMRs of university students disaggregated by suicide motive and gender, a multiple regression analysis with robust s.e. was conducted with gretl (version 2022b for Windows; see https://gretl.sourceforge.net/win32/index_es.html).

A portion of the SMRs for suicides associated with the 52 subcategorised suicide motives, disaggregated by gender and age, were excluded from this study because these SMRs were small (values of <2.5%) and highly variable, which an ITSA impossible. These were no missing data in SSNPA or VSR. The total numbers of suicides of high school, university and special vocational school students during 2007–2022 in Japan are summarised in Supplementary Tables 2–4, respectively.

This study adhered to the Strengthening the Reporting of Observational Studies in Epidemiology (STROBE) reporting guidelines. The medical ethics review committee of Mie University waived the requirements for informed consent and ethical approval because the study used data that are available from publicly accessible governmental databases.

## Results

### SMRs of students and non-student youths during 2007–2019

**Fig. 1:**
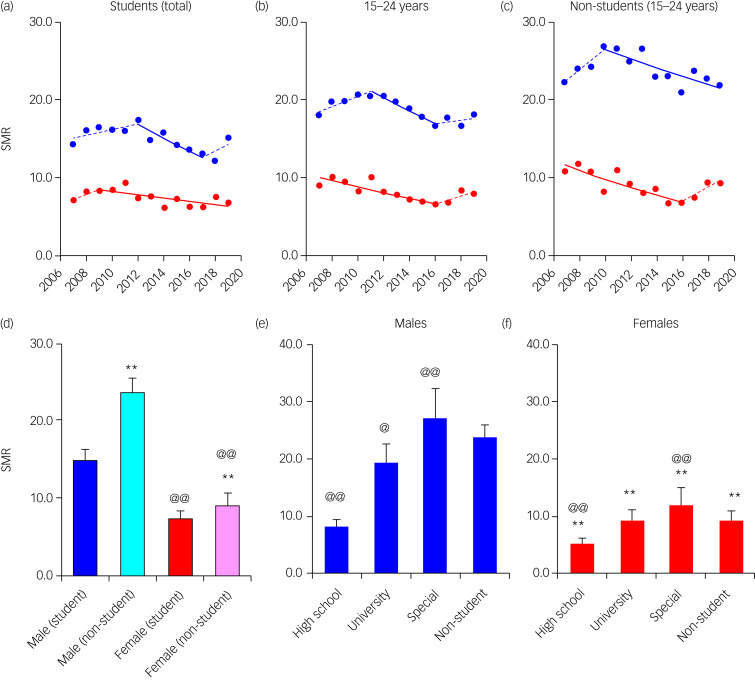
SMRs disaggregated by gender (males and females) and students (high school, university, special vocational school and non-students) in youth during 2007–2019. Trends of SMRs of students, total and non-students in youth (15–24 years) analysed with joinpoint regression analysis (JPRA) shown in Figs 1(a), 1(b) and 1(c), respectively. The ordinate and abscissa indicate the SMRs per 100 000 population and years, respectively. Solid blue and red lines indicate the significant trends of SMRs of males and females detected by JPRA, respectively. Dotted blue and red lines indicate the non-statistically significant trends of SMRs of males and females detected by JPRA, respectively. The mean ± s.d. of SMRs of males and females of students and non-students in youth during 2007–2019 are indicated in Fig. 1(d). **P < 0.01 and @@*P* < 0.01 indicate the statistically significant differences compared with students and males by linear mixed-effects models with Scheffe's *post hoc* test, respectively. The mean ± s.d. of SMRs of students in high school, university, special vocational school (special) and non-students in males (Fig. 1(e)) and females (Fig. 1(f)) during 2007–2019 are indicated in Figs 1(e) and 1(f), respectively. **P < 0.01 indicates the statistically significant differences compared with males by linear mixed-effects models with Scheffe's *post hoc* test. @*P* < 0.05 and @@*P* < 0.01 indicate the statistically significant differences compared with non-students by linear mixed-effects models with Scheffe's *post hoc* test. SMR, suicide mortality rate.

The SMRs of non-student youth were larger than those of students (total students in high school, university and special vocational school); however, unusually, the SMRs of special vocational school students were larger than those of non-students (Fig. 1). The male SMRs of all groups were greater than female SMRs (Fig. 1). The JPRA detected the joinpoints of SMRs of male students (2012 and 2017) and female non-students (2017) around the switching periods from first to second or from second to third GPSPP periods; however, the joinpoints of female students and male non-students were 2009 and 2010, respectively (Fig. 1). Therefore, the JPRA could not detect consistent temporal relationships between SMR fluctuation patterns and GPSPP periods.

### Major motives in student SMRs during 2007–2022

The student populations (mean ± SD) were as follows: high school (males/females: 1 648 274 ± 67 520/1 614 828 ± 68 032), university (males/females: 1 652 689 ± 32 721/1 229 142 ± 57 484) and special vocational school (males/females: 289 274 ± 9250/357 635 ± 12 445). Total suicides from 2007 to 2022 reported in the SSNPA were as follows: high school (males/females: 2376/1566), university (males/females: 5179/1880) and special vocational school (males/females: 1233/690) (Supplementary Tables 2–4).

Male SMRs were larger than female SMRs, and SMRs increased in the order of high school, university and special vocational school (Supplementary Fig. 1(a)). Among males, school-related motives were the leading cause of suicide, followed by health- and family-related motives, whereas in females, health-related motives were the leading cause, followed by school- and family-related motives (Supplementary Fig. 1(a)).

**Fig. 2:**
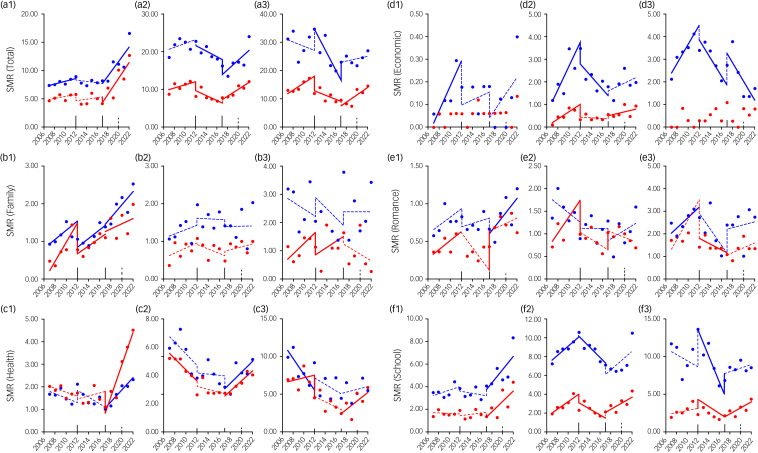
Fluctuations of student SMRs of suicides associated with six major motives during 2007–2022 in Japan, analysed by ITSA. Fluctuations of trends of SMRs of students in high school (a1–f1), university (a2–f2) and special vocational school (a3–f3), for suicides associated with total (a1–a3), family-related (b1–b3), health-related (c1–c3), economic-related (d1–d3), romance-related (e1–e3) and school-related (f1–f3) motives are represented. The ordinates and abscissas indicate the annual SMRs (per 100 000 population) and years, respectively. Blue and red circles indicate the observed annual SMRs of males and females, respectively. Solid blue and red lines indicate the significant trends of SMR of males and females detected by ITSA, respectively. Dotted blue and red lines indicate the non-statistically significant trends of SMR of males and females detected by ITSA, respectively. SMRs of suicides associated with employment-related motives were not indicated because it was impossible to analyse this with ITSA. ITSA, interrupted time-series analysis; SMR, suicide mortality rate.

Most SMRs with significant changes detected in the first GPSPP period showed a significant increase, except for health-related motives among female university and male special vocational school students, which significantly decreased (Fig. 2 and Supplementary Fig. 2). In contrast, most SMRs with significant changes detected in the second GPSPP period showed a significant decrease. Notably, family-related motives significantly increased in high school students of both genders (Fig. 2 and Supplementary Fig. 2). Most SMRs with significant changes detected in the third GPSPP period showed a significant increase. However, economy-related motives decreased in male special vocational school students. In particular, family-related motives consistently increased in high school students of both genders during 2007–2022 (Fig. 2 and Supplementary Fig. 2).

### School-related motives

Underachievement was the leading cause of male suicides, followed by worrying about the future and entrance examinations (Supplementary Fig. 1(b)). Underachievement was also the leading cause of female SMRs among special vocational school students, followed by worrying about the future and conflict with classmates. In contrast, among the SMRs of female high school and university students, worrying about the future was the leading motive, followed by underachievement and conflict with classmates (Supplementary Fig. 1(b)).

**Fig. 3:**
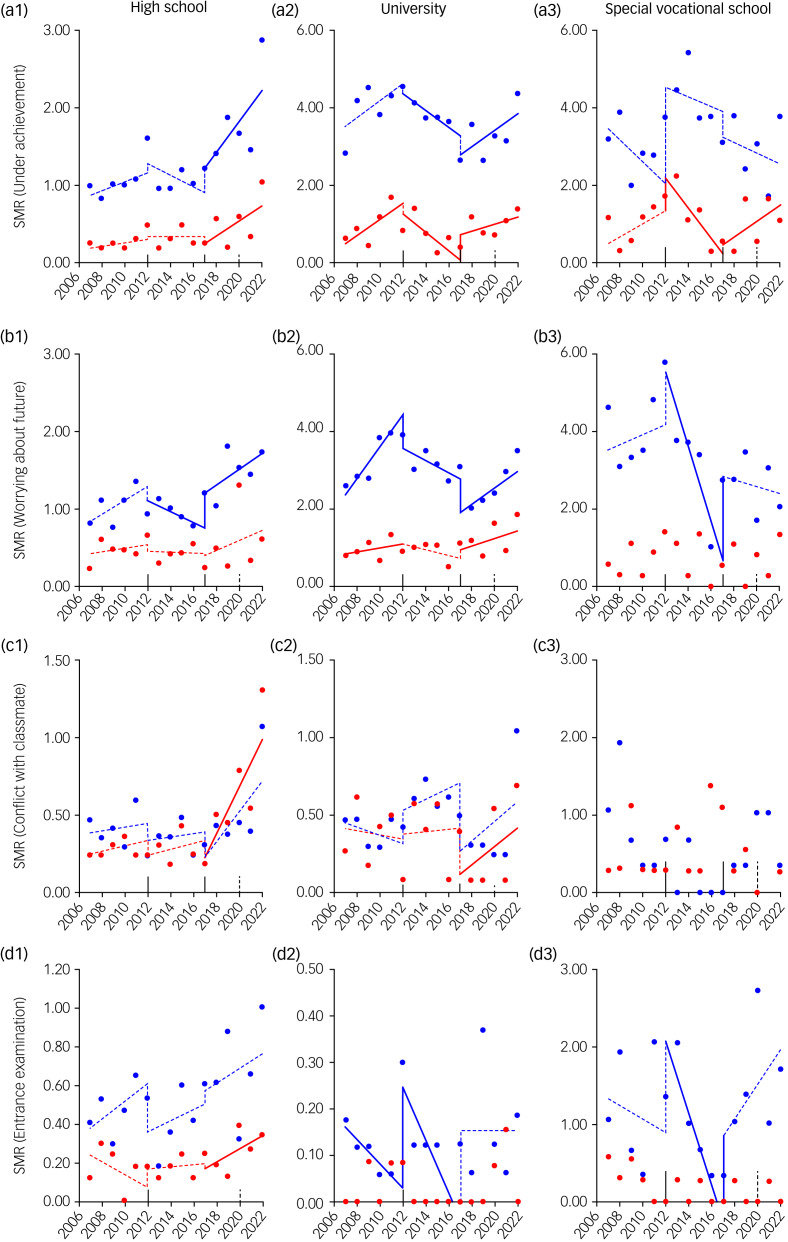
Fluctuations of student SMRs for suicides associated with subcategories in school-related motives during 2007–2022 in Japan, analysed by ITSA. Fluctuations of trends of SMRs of students in high school (a1–d1), university (a2–d2) and special vocational school (a3–dD3), for suicides associated with underachievement (a1–a3), worrying about the future (b1–b3), conflict with classmate (c1–c3) and entrance examination (d1–d3) are represented. The ordinates and abscissas indicate the annual SMRs (per 100 000 population) and years, respectively. Blue and red circles indicate the observed annual SMRs of males and females, respectively. Solid blue and red lines indicate the significant trends of SMR of males and females detected by ITSA, respectively. Dotted blue and red lines indicate the non-statistically significant trends of SMR of males and females detected by ITSA, respectively. ITSA, interrupted time-series analysis; SMR, suicide mortality rate.

In high school, during the first and second GPSPP periods, most school-related motives did not change; a notable exception was worrying about the future among males, which significantly decreased (Fig. 3 and Supplementary Fig. 3). However, during the third GPSPP period, SMRs for male suicides associated with underachievement and worrying about the future, and female suicides associated with underachievement, conflict with classmates and entrance examination, increased. In university students, consistent trends could not be detected in male SMRs, whereas female suicides associated with underachievement and worrying about the future increased during the first and third GPSPP period. In special vocational schools, the SMRs showed no consistent trends in either gender (Fig. 3 and Supplementary Fig. 3).

### Health-related motives

**Fig. 4:**
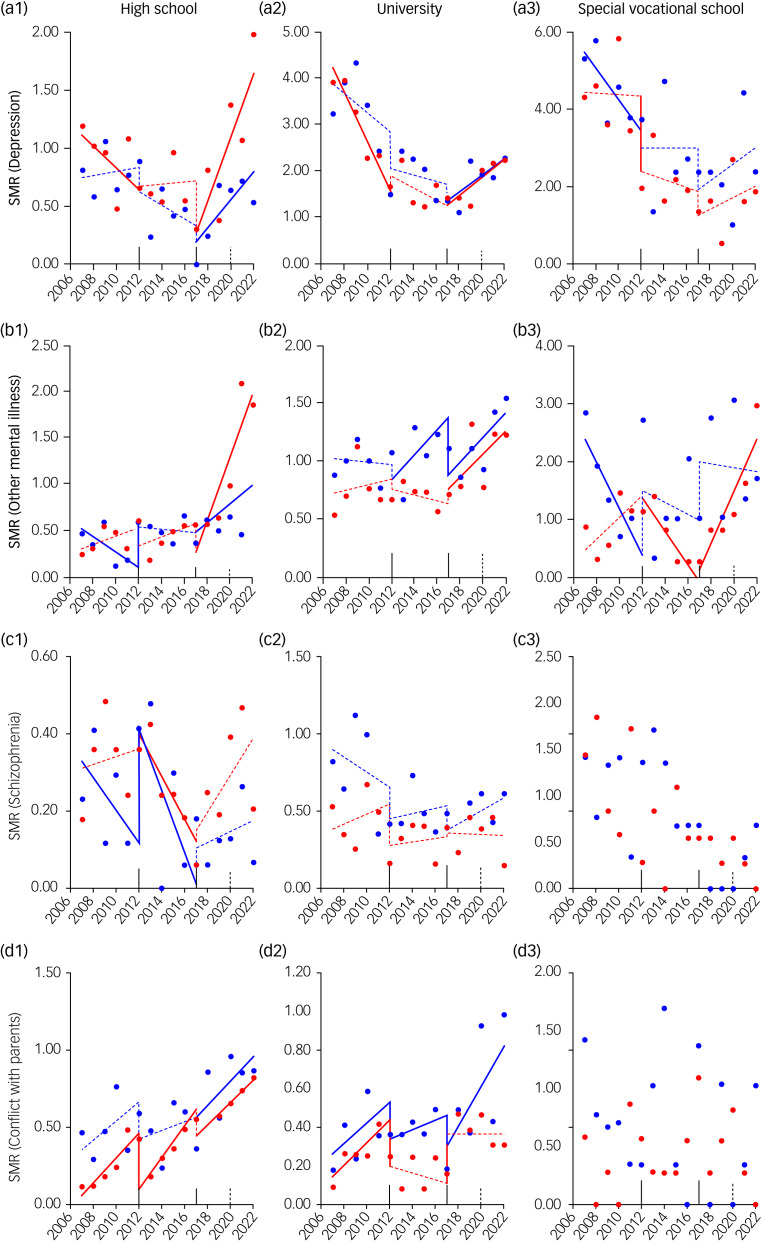
Fluctuations of student SMRs for suicides associated with subcategories in health-related and family-related motives during 2007–2022 in Japan, analysed by ITSA. Fluctuations of trends of SMRs of students in high school (a1–d1), university (a2–d2) and special vocational school (a3–d3) for suicides associated with depression (a1–a3), other mental illness (b1–b3), schizophrenia (c1–c3) and conflict with a parent (d1–d3) are represented. The ordinates and abscissas indicate the annual SMRs (per 100 000 population) and years, respectively. Blue and red circles indicate the observed annual SMRs of males and females, respectively. Solid blue and red lines indicate the significant trends of SMR of males and females detected by ITSA, respectively. Dotted blue and red lines indicate the non-statistically significant trends of SMR of males and females detected by ITSA, respectively. ITSA, interrupted time-series analysis; SMR, suicide mortality rate.

Depression was the leading cause of suicide in both genders, followed by other mental illness and schizophrenia (Supplementary Fig. 1(c)). In female high school and university students, the SMRs for suicides associated with depression and other mental illnesses did not change to a statistically significant extent during the second GPSPP period, but drastically increased during the third GPSPP period (Fig. 4 and Supplementary Fig. 3). Among female special vocational school students, the SMR for suicides associated with other mental illnesses decreased during the second GPSPP period, but conversely increased during the third GPSPP period; however, the SMR for suicides associated with depression did not change to a statistically significant extent between the second and third GPSPP periods (Fig. 4 and Supplementary Fig. 3). In male high school and university students, the SMR for suicides associated with depression and other mental illnesses significantly increased during the third GPSPP period. However, the SMR of male special vocational students for suicides associated with depression and other mental illness did not change during the third GPSPP period (Fig. 4 and Supplementary Fig. 3).

### Family-related motives

SMRs for suicides associated with conflict with parents consistently increased in female high school students and male university students (Fig. 4 and Supplementary Fig. 3). The SMR for suicides associated with conflict with parents showed a non-significant increase in male high school students during the first and second GPSPP periods, followed by a significant increase during the third GPSPP period. Among female university students, the SMR for suicides associated with conflict with parents increased during the first GPSPP period, but did not change during the second or third GPSPP period; however, during the third GPSPP period, the SMR significantly increased before the COVID-19 pandemic and significantly decreased after the pandemic (Fig. 4 and Supplementary Fig. 3). The SMRs for suicides associated with severe reprimand increased in high school students of both genders during the first GPSPP period, but did not significantly change during the second or third GPSPP period. However, during the third GPSPP period, SMRs for suicides associated with severe reprimand increased in high school students of both genders after the pandemic (Fig. 4 and Supplementary Fig. 3).

### Effect of grade repetition on SMRs for university students

A multiple regression analysis detected the consistent impact of 2 years of grade repetition on SMRs of suicides associated with underachievement in both genders (Supplementary Table 5). However, no consistent impact of grade repetition on SMRs for suicides with other motives was noted in either gender. In males, the SMRs of suicides associated with worrying about the future were positively associated with 1 year of grade repetition. In contrast, in females, these SMRs were positively associated with 3 years of grade repetition and negatively associated with 1 year of grade repetition (Supplementary Table 5). The temporal gender gap regarding SMRs for suicides associated with worrying about the future suggests that the psychological background underlying this differed between the genders. Long-term exposure may induce impulsivity and exhaustion, resulting in increased suicide risk in males and females, respectively.^19,20^ Contrary to our expectations, longer grade repetition (4 or more years) was negatively associated with SMRs for suicides associated with health-related motives, including depression in both genders and schizophrenia in males (Supplementary Table 5).

## Discussion

### Effects of educational status

This study demonstrated that SMRs among non-student youth (age 15–24 years)^4^ were higher than those for total students; however, the SMRs of special vocational students of both genders were larger than those for non-student youth. The ‘Survey on Undesirable Behaviour and School Nonattendance of Students’ (https://www.e-stat.go.jp/en/statistics/00400304) and the ‘2017 White Paper on Education, Culture, Sports, Science and Technology’ (https://warp.ndl.go.jp/info:ndljp/pid/11509864/www.mext.go.jp/b_menu/hakusho/html/hpab201701/1417254.htm) published by MEXT, reported that special vocational schools mainly provide opportunities to acquire educational qualifications and specialised skills. Therefore, the school attendance time/period is shorter, and the student support system needed in special vocational schools is also relatively smaller compared with high schools and universities. Furthermore, the rate of financial support from parents is lower and the rate of employment after graduate of special vocational school is higher compared with university students (most high school students are financially dependent on their parents). Therefore, required academic achievement and individual psychological and economic independence appear to play important roles in suicide among youth, but these factors differ greatly among high school, university and special vocational school students. Furthermore, considering that the suicide prevention measures implemented for students in GPSPP consisted of protective policies for the socially vulnerable under the protection of adults via enhanced welfare/safety nets and protection systems for vulnerabilities in schools,^2^ this study suggests that GPSPP-based suicide prevention programmes in high schools and universities have at least partially contributed to suicide prevention in the student population. Indeed, a systematic review in the Western Pacific region, including the Philippines, China, Cambodia and Malaysia, suggested that the effective categories in school health services were wide community engagement, youth focus and participation, delivery of high-quality comprehensive care, and effective governance and administrative systems.^21^ GPSPP has already listed these four categories as major priorities.^2^

### Impact of GPSPP on SMRs of students

The first GPSPP period (2007–2012) initially listed suicide prevention of students/children as subcategories in nine priority major categories. The second GPSPP period (2012–2017) added consultation programmes for abused/bullied children as subcategories. The third GPSPP period (2017–2022) additionally listed new priority major categories: ‘development of a suicide prevention programme for children/adolescents’, involving the enhancement of support systems for bullied/abused student/children in schools/communities, and ‘enlightenment of how to represent signs for seeking support against high-risk student/children’.^9^

It is well-known that bullying and physical and/or verbal abuse are risk factors for suicide in students and youth.^22^ When student suicides occur because of abuse or bullying, an independent committee, composed of specialists in education, psychiatrists, politicians and other academics, must investigate the details of the causes; the details are widely reported in the mass media, resulting in national attention. Furthermore, the ‘Survey on Undesirable Behaviour and School Nonattendance of Students’ published by MEXT reported an eight-fold increase in the incidence of suicide associated with bullying/abuse in Japan from 2007 to 2021. However, the SMR of suicides associated with abuse or bullying in students accounted for <1% of the SMR for students of both genders, and no significant increase was observed from 2007 to 2021. Considering the drastically increasing rates of bullying/abuse, it is considered that GPSPP contributed to the prevention of suicides among students who experience bulling or abuse (Supplementary Tables 2–4).

The present study could not confirm that SMRs decreased among students during the whole observation period (2007–2022). Indeed, during the first GPSPP period, the SMRs of high school and university students did not decrease, and although the SMRs of high school and university students of both genders decreased during the second GPSPP period, they unexpectedly and drastically increased during the third GPSPP period. Therefore, the fluctuation patterns of the SMRs of high school and university students were completely independent of the policy objectives and reasons of the GPSPP. However, this study detected similarities in SMRs for suicides associated with school- (underachievement) and health- (depression and other mental illness) related motives between high school and university students of both genders during the third GPSPP period. Furthermore, SMRs for suicides associated with conflict with parents in female high school students and male university students consistently increased throughout the observation period.

### Candidate underlying mechanisms of increasing student SMRs

The increasing trend in SMRs for suicides associated with three suicide motives, specifically family-related (conflict with parent), school-related (underachievement/worrying about the future) and health-related (mental illnesses) motives, from the late 2010s suggests the possibility that internalising symptoms/disorders may have fundamental or underlying roles in increasing student suicides. Parents, educators and students themselves expect to perform well in school, since performing well in school leads to a range of positive life outcomes, including happiness, physical/mental health and occupational status.^23,24^ To achieve good educational performance, parents tend to adopt attitudes with low levels of emotion and high levels of parental control or overprotection. Both the perceived pressure of schoolwork and parental educational style (low care and high protection) are considered to be risk factors for mental illness, such as internalising symptoms/disorders in students/adolescents, leading to increased suicide risk.^24–27^ A negative vicious cycle among ‘perceived schoolwork pressure’, ‘parental educational style (low care and high protection)’ and ‘internalising symptom/disorders’ is considered to be developing in Western countries.^25–27^ Additionally, the declining birth rate and population ageing in East Asian regions, including China, South Korea and Japan, have resulted in an increase in single-child families.^28^ The prevalence of emotional/behavioural problems, including internalising symptoms, of firstborn children with siblings is relatively lower compared with children without siblings.^29^ Therefore, the present results suggest that internalising symptoms/disorders may have been rapidly increasing in Japan since the late 2010s, as a result of the addition of the declining birth rate to this negative vicious cycle.^17^

Indeed, the ‘Patient Survey’ published by the MHLW (https://www.e-stat.go.jp/en/statistics/00450022) reported that the prevalence of internalising disorders in individuals of 10–24 years of age increased in 2020 compared with 2017.^17^ Furthermore, it has been reported that the internalising symptoms exhibited by students differ depending on their educational performance.^30^ Depression was the predominant symptom in students with low educational performance, whereas anxiety (or uncontrolled worry) was the predominant symptom in students with good educational performance.^30^ Additionally, students with mental health issues infrequently seek support, which is considered a major barrier to the implementation of suicide prevention programmes.^2,31^ Therefore, to effectively support students who are suffering from underachievement or worried about their future, their parents, schools and community need to make efforts to observe, detect and implement measures more widely.

Perceived schoolwork pressure and parental educational style (low care and high protection) might be unavoidable for parents and educators to achieve better life outcomes for their children. Therefore, recent trends in ways of achieving better life outcomes for children probably contribute to internalising symptoms and increasing youth suicide in Japan.^17^ The suppression and improved control of these current trends of perceived schoolwork pressure and parental educational style (low care and high protection) seem attractive interventions for the suppression of increasing student suicides; however, interventions such as controlling schoolwork pressure and/or parental educational style are associated with risks, such as violating the basic dignity of individuals (students and parents) and unexpected negative effects on individual sociopsychological development.

### Mental health support and suicide prevention programmes in school

Taken together with the recent increasing prevalence of psychiatric disorders in the youth population, it can be speculated that the prevalence of mental illnesses among students is also increasing. The age at onset of internalising symptoms/disorders was approximately 15 years of age, and their prevalence increased dramatically after the transition from middle school to high school.^32^ Furthermore, increasing SMRs of suicides associated with mental illnesses among high school students preceded those among university students, suggesting that the increasing prevalence of internalising symptoms/disorders possibly plays an important role in the increasing SMR of the youth population. In Japan, the Act for Eliminating Discrimination against Persons with Disabilities was implemented in 2016.^33^ Mental health support services in schools (elementary, junior high, high school and university) have mainly implemented enhancement of mental health support staff to prevent mental health impairment among students.^33^

Although it is impossible to confirm the exact numbers of students who developed mental illness when attending school and newly registered students who suffered from mental illness, taken together with the recent increase in the prevalence of psychiatric disorders in the youth population, it can be speculated that there has been an increase in the number of university students who are suffering from internalising symptoms/disorders. Therefore, we hypothesised that the lack of a support system for newly registered university students who are already suffering from mental illnesses was partly responsible for the recent increase in student suicides.

The association of grade repetition and suicide motives, such as school- and health-related motives, with SMRs among university students has important implications regarding targets for student support at the university level. Underachievement and grade repetition are serious challenges that university students must overcome.^33,34^ For students who overcame grade repetition owing to underachievement within 1 year, underachievement was not a risk factor for suicide, whereas multiple-grade repetition (2 years) was found to be a major risk factor for suicide in both gender. In males, SMRs for suicides associated with by worrying about the future were positively associated with 1 year of grade repetition, whereas in females, SMRs were positively associated with 3 years of grade repetition (Supplementary Table 5). In contrast, longer-grade repetition (four or more years) was negatively associated with SMRs for suicides associated with health-related motives, including depression and schizophrenia. Therefore, the postponement of this reprieve exerts social protection for some university students who repeat grades and have a mental illness that requires long-term treatment before their return to university.

How can schools support students facing these difficult/complicated situations? In addition to providing opportunities to receive professional education and mental support for preventing mental health impairments, the campus may contribute to supporting mental health and suicide prevention by developing/increasing sociopsychological and socioeconomic professionals, who can support students facing stigma and frustration (e.g. students with various problems such as those mentioned above, students who repeat grades and students who drop out).

### Effects of the COVID-19 pandemic

During the COVID-19 pandemic, social restriction measures for controlling the spread of COVID-19 resulted in students having limited access to recreational activities and educational services, with a great amount of time spent at home. It has been hypothesised that the lifestyle transformations of students during the pandemic have had both adverse (increasing conflicts with family, reducing opportunities for stress coping and education) and protective (promotion of strong intrafamilial cohesion by spending more time together, reducing schoolwork pressures) effects on the risk of suicide among students.^17,35^ Although the difference in 2022 was not statistically significant, SMRs for suicides associated with certain motives (health-related: depression/other mental illnesses; school-related: worrying about the future/conflict with classmates) drastically increased. This suggests that the normalisation of lifestyles after restrictions possibly further promoted these traditional risk factors for suicides. In other words, the increasing SMRs were affected by maladaptation to the restrictions in the initial stage of the pandemic, and the return to normal in the late stage of the pandemic.

### Limitations

This study has several strengths, including its national-level, population-based analysis of a government database; its youth population (15–24 years), including students disaggregated by suicide motives; and the coverage of a wide and recent period (2007–2022). However, this study also has several limitations. The second half of the third GPSPP period (2017–2022) overlapped with the COVID-19 pandemic. During the pandemic, students had limited access to recreational activities and educational services, with a great amount of time spent at home because of restriction measures. These lifestyle transformations of students during the pandemic may have increased the suicide risk of students by increasing conflicts with family and reducing opportunities for stress coping and education; and decreased suicide risk via the promotion of strong intrafamilial cohesion by spending more time together.^17,35^ Therefore, to analyse the effects of the pandemic on SMRs, the pandemic outbreak period (2020) was added into ITSA analysis as the additional intervention period; however, it was impossible to exclude the effects of the pandemic from the analysis of the impact of the third GPSPP period on SMRs.

The second limitation is that in this study, each GPSPP period as an intervention period was analysed by ITSA; however, there may have been time lags until the effects of newly implemented policies were realised in the next GPSPP. In particular, policies that use enlightenment can be expected to have an early effect, whereas policies that require specialists or changes/improvements in social structure, which require financial expenditure, are likely to take longer. Therefore, it is necessary to analyse the relationship between prefectural SMRs and fiscal expenditures corresponding to GPSPP, using fixed-effects hierarchical linear models and/or vector autoregression analyses. However, as of now, the latest data on financial expenditures for each prefecture in Japan are from 2019; thus, this should be investigated in future studies.

The third limitation is the lack of detailed data on ‘other mental illnesses’, one of the most affected motives and risk factor for suicide in the younger population detected by this study. The WHO listed depression, anxiety, eating disorders and heavy episodic drinking as risk factors for adolescent suicide.^1^ Although it is controversial, global imputation modelling survey data on self-reported mental health problems have demonstrated a recent increase in the prevalence of mood and anxiety symptoms and substance misuse.^35^ Therefore, elucidating the actual conditions of other mental illnesses that influence youth suicide could greatly contribute to the improvement of suicide prevention programmes in Japan.

Finally, to eliminate subjectivity as much as possible, police investigate suicide motives based on evidence, suicide notes, official documentation (e.g. medical certificates and clinical recordings) and testimony from the victim's family; however, collecting suicide motives from direct victims is impossible. Therefore, suicide numbers disaggregated by suicide motives in the SSNPA may be over- or underestimated because of potential bias. Despite these limitations, the SSNPA is considered to be the most reliable governmental suicide database in Japan, since data are collected by the NPA according to methods that are regulated by law.

## Supporting information

Matsumoto et al. supplementary material Matsumoto et al. supplementary material

## Data Availability

All raw data are publicly available to any persons via Japanese national databases from the Basic Data on the SSNPA published by the NPA (https://www.npa.go.jp/publications/statistics/safetylife/jisatsu.html), the VSR published by the MHLW (https://www.mhlw.go.jp/stf/seisakunitsuite/bunya/hukushi_kaigo/seikatsuhogo/jisatsu/index.html), the Regional Statistics Database published by the System of Social and Demographic Statistics of the Statistics Bureau of the Ministry of Internal Affairs and Communications (https://www.e-stat.go.jp/en/regional-statistics/ssdsview), and the School Basic Survey published by the MEXT (https://www.e-stat.go.jp/en/statistics/00400001).
