# Supplementary material for: Impact of the Japanese Government's ‘General Principles of Suicide Prevention Policy’ on youth suicide from 2007 to 2022
Source: BJPsych Open. 2023 Dec 19;10(1):e16. doi: 10.1192/bjo.2023.616 (PMC10755549; doi:10.1192/bjo.2023.616)
Supplement: Matsumoto et al. supplementary material [file S2056472423006166sup001.pdf]

Supplemental Data

Supplementary Table s1. Priority major categories of governmental "General Principles of Suicide Prevention Policy" (GPSPP) in 1st (2007-2012), 2nd (2012-2017) and 3rd (2017-2022) periods

Supplementary Table s2. Suicide numbers of high school students disaggregated by motives and gender during 2007-2022.

Supplementary Table s3: Suicide numbers of university students disaggregated by motives and gender during 2007-2022.

Supplementary Table s4: Suicide numbers of special vocational school students disaggregated by motives and gender during 2007-2022.

Supplementary Fig.s1: SMRs caused by suicidal motives of high school, university and special vocational school students of males and females during 2007–2022.

Supplementary Fig.s2: Trends of SMRs caused by 6 major suicidal motives during 2007-2022.

Supplementary Fig.s3: Trends of SMRs caused by suicidal motives in school-, health- and family-related problems during 2007-2022.

Supplementary Table s5: : Effects of grade repetitions on SMRs of university students caused by suicidal motives

Supplementary Table s1: Priority major categories of governmental "General Principles of Suicide Prevention Policy" (GPSPP) in 1st (2007-2012), 2nd (2012-2017) and 3rd (2017-2022) periods

1st PSPP (2007-2012)	2nd PSPP (2012-2017)	3rd PSPP (2017-2022)
**1.Clarify the actual situation of the suicide**	**1.Clarify the actual situation of the suicide**	**1. Clarify the actual situation of the suicide**
Investigation of the actual situation of suicide	Investigation of the actual situation of suicide	
Enhancement of information of reporting system	Enhancement of information of reporting system	
Survey about the actual situation and the support for suicide attempter, bereaved family		
Survey about the suicide prevention of student	Survey about the suicide prevention of student	
Investigations of pathophysiology of mood disorder	Investigations of pathophysiology of mood disorder	
promotion of utilization of existing materials		
		Innovative suicide research promotion programme
		(investigative research, verification and utilization of survey findings)
		Collection, organization and provision of information on advanced initiatives
		Survey of children/adolescent suicides
		Coordination with the cause-of-death investigation system
		Accumulation, organization and analysis of information related to suicide prevention
2. Promoting awareness and observation by each individual	2. Promoting awareness and observation by each individual	2. Promoting awareness and observation by each individual
Enlightenment of suicide prevention programme week	Enlightenment of suicide prevention programme week	Enlightenment of suicide prevention programme week
Implementation of education about suicide prevention among student	Implementation of education about suicide prevention among student	Implementation of education about suicide prevention among student
Enlightenment of mood disorder	Enlightenment of mood disorder	Enlightenment of mood disorder
	Dissemination of knowledge about suicide and suicide-related events	Dissemination of knowledge about suicide and suicide-related events
		Enlightenment of importance about SOS
**3. Development of Professionals for suicide prevention**	**3. Development of Professionals for suicide prevention**	**3. Development of Professionals for suicide prevention**
Psychiatric education for general physicians	Psychiatric education for general physicians	Psychiatric education for general physicians
Enlightenment of suicide and suicide prevention programme to educationists	Enlightenment of suicide and suicide prevention programme to educationists	
Implementation of training for long-term care support specialists	Implementation of training for long-term care support specialists	
Improving the quality of community/occupational health staffs		Improving the quality of community/occupational health staffs
Implementation of training for regional welfare officers and children's commissioners		
Development of regional leaders		
Improving the quality of counsellors related to social		
Improving the quality of public staffs who contact with bereaved families.		
Development of mental health supportive system for staffs of suicide prevention programme		
Developing training materials		
	Improving the quality of counsellors related to overloaded debts, unemployment and economic hardship	
	Development of gatekeeper	Development of gatekeepers
		Support for supporters including family members and acquaintances
		Promotion of education of suicide prevention in university
**4. Promoting mental health**	**4. Promoting mental health**	**4. Promoting mental health**
Promotion of occupational mental health supports	Promotion of occupational mental health supports	Promotion of occupational mental health supports
Development of mental health support system in community	Development of mental health support system in community	Development of mental health support system in community
Development of mental health support service in schools	Development of mental health support service in schools	Development of mental health support service in schools
	Enhancement of mental care for victims of disasters and support their life reconstruction	Enhancement of mental care for victims of disasters and support their life reconstruction
**5. Enhancement of psychiatric care system**	**5. Enhancement of psychiatric care system**	**5. Enhancement of psychiatric care system**
Development of psychiatric co-medical staffs	Development of psychiatric co-medical staffs	
Increasing consultation rate of depression	Increasing consultation rate of depression	
Enhancement of measures for high-risk individuals with mental illnesses other than depression	Enhancement of measures for high-risk individuals with mental illnesses other than depression	
Support for patients with chronic diseases	Support for patients with chronic diseases	
Development of mental health support system for children/adolescences		
		Enhancement of measures for high-risk individuals with mental illnesses other than depression
		Enhancement of measures for high-risk individuals with mental illnesses, including depression, schizophrenia, several dependencies
		Development of human resources responsible for mental health medical welfare services
**6. Preventing suicide through social cooperation**	**6. Preventing suicide through social cooperation**	**6. Preventing suicide through social cooperation**
Enhancement of consultation system in community	Enhancement of consultation system in community	Enhancement of consultation system in community
Enhancement of consultation system for overloaded debts	Enhancement of consultation system for overloaded debts	Enhancement of consultation system for overloaded debts
Enhancement of counselling system for unemployed people	Enhancement of counselling system for unemployed people	Enhancement of counselling system for unemployed people
Enhanced support for caregivers	Enhanced support for caregivers	Enhanced support for caregivers

Disseminate WHO guideline to the mass media	Disseminate WHO guideline to the mass media	Disseminate WHO guideline to the mass media
Preventing suicide in bullied children	Preventing suicide in bullied children	Preventing suicide in bullied children
	Enhancement of for telephone counselling service for bullying children	Enhancement of for telephone counselling service for bullying children
Preventing threats of suicide in Internet	Preventing threats of suicide in Internet	Preventing threats of suicide in Internet
	Responding to suicide notice using Internet	Responding to suicide notice using Internet
Enhancement of information provision for solving legal problems		
Restrictions about hazardous places and chemicals		
Consultation for management		
	Enhancement of support systems for victims of child abuse and sex crime	Enhancement of support systems for victims of child abuse and sex crime
	Enhancement of support system for economic hardship	Enhancement of support system for economic hardship
		Enhancement of consultation services using internet and SNS
		Development of diverse consultations and strengthening outreach
		Disseminate information sharing necessary for cooperation among related organizations
		Promoting of places for stay contributing to suicide prevention
		Enhancement of support for expectant and nursing mothers
		Enhancement of support for LGBT
**7. Preventing repeated suicidal behaviours in suicide attempters**	**7. Preventing repeated suicidal behaviours in suicide attempters**	**7. Preventing repeated suicidal behaviours in suicide attempters**
Improving the liaison consultation psychiatric system in medical facilities	Improving the liaison consultation psychiatric system in medical facilities	
Support for observation/protection by family members	Support for observation/protection by family members	
		Enhancement of comprehensive support systems for suicidal attempters via collaboration among medical and community
		Development of regional medical centre for prevention repeated suicide attempt behaviours
		Development of safety places
		Development of supporting systems in schools and workplaces
**8. Enhancement of support for bereaved families of suicide victim**	**8. Enhancement of support for bereaved families of suicide victim**	**8. Enhancement of support for bereaved families of suicide victim**
Supporting the self-help groups for bereaved families of suicide victims	Supporting the self-help groups for bereaved families of suicide victims	Supporting the self-help groups for bereaved families of suicide victims
Development of supporting systems for bereaved families of suicide victim	Development of supporting systems for bereaved families of suicide victim	
Development of brochure for bereaved families of suicide victim	Development of brochure for bereaved families of suicide victim	Development of brochure for bereaved families of suicide victim
Enhancement of support for bereaved children of suicide victim		Enhancement of support for bereaved children of suicide victim
		Improving the quality of governmental staffs contact with bereaved families
		Enhancement of provision of information for comprehensive support bereaved families of suicide victims
		Development of supporting systems for bereaved families of suicide victims in schools and workplaces
**9. Enhancement of cooperation with private organizations**	**9. Enhancement of cooperation with private organizations**	**9. Enhancement of cooperation with private organizations**
Development of human resource for suicide prevention	Development of human resource for suicide prevention	Development of human resource for suicide prevention
Establishment of regional cooperation system	Establishment of regional cooperation system	Establishment of regional cooperation system
Support for telephone counselling service of private organizations	Support for telephone counselling service of private organizations	Support for telephone counselling service of private organizations
Support for pioneering/trial efforts by private organizations	Support for pioneering/trial efforts by private organizations	Support for pioneering/trial efforts by private organizations
		**10. Development of suicide prevention programme for child/adolescent**
		Enhancement of suicide prevention of suicide among bullying children
		Enhancement of support for students
		Education how to request supports
		Enhancement of support system for children
		Enhancement of support system for adolescents
		Enhancement of support young generation based on their specific features
		Support for acquaintances
		**11. Enhancement of prevention of suicide caused by employment-related causes**
		Enhancement of long working hours promotion of mental health in workplace
		Harassment prevention measures
		**12. Enhancement of regional suicide prevention programmes**
		Development of regional suicidal profile and political package for regional suicide prevention programmes
		Development of guidelines for regional suicide prevention programmes
		Enhancement of regional suicide prevention centres
		Promoting the establishment of dedicated departments and professional staffs for suicide prevention programmes in regional governments

Red painted lists were newly added priority categories.

Supplementary Table s2: Suicide numbers of high school students disaggregated by motives and gender during 2007-2022.

	Males						Females					
	Total	(	min	/	max	)	Total	(	min	/	max	)
**Total**	2376	(	249	/	123	)	1566	(	185	/	66	)
**Family problems**	369	(	38	/	15	)	270	(	29	/	6	)
Conflict with parent	153	(	15	/	4	)	113	(	14	/	2	)
Marital conflict	0	(	0	/	0	)	2	(	1	/	0	)
Conflict with other family members	35	(	7	/	0	)	43	(	5	/	0	)
Death of family	16	(	2	/	0	)	15	(	3	/	0	)
Hopeless for family	16	(	3	/	0	)	10	(	3	/	0	)
Severe verbal reprimand	95	(	15	/	2	)	58	(	8	/	1	)
Stress of raising children	0	(	0	/	0	)	0	(	0	/	0	)
Physical and/or verbal abuse	4	(	1	/	0	)	4	(	2	/	0	)
Exhaustion from caring for infirm family	0	(	0	/	0	)	0	(	0	/	0	)
Other family-related problems	50	(	7	/	1	)	25	(	3	/	0	)
**Health problems**	430	(	36	/	17	)	523	(	66	/	18	)
Physical illness	52	(	6	/	1	)	31	(	5	/	0	)
Depression	155	(	18	/	0	)	224	(	29	/	5	)
Schizophrenia	54	(	8	/	0	)	75	(	8	/	1	)
Alcoholism	0	(	0	/	0	)	0	(	0	/	0	)
Drug abuse	3	(	2	/	0	)	3	(	1	/	0	)
Other mental illness	132	(	18	/	2	)	168	(	31	/	3	)
Physical disability	8	(	3	/	0	)	7	(	2	/	0	)
Other health-related problems	26	(	6	/	0	)	15	(	3	/	0	)
**Economic problems**	34	(	6	/	0	)	14	(	2	/	0	)
Bankruptcy	0	(	0	/	0	)	0	(	0	/	0	)
Business struggling	0	(	0	/	0	)	0	(	0	/	0	)
Unemployment	0	(	0	/	0	)	0	(	0	/	0	)
Inability to find employment	14	(	4	/	0	)	4	(	2	/	0	)
Economic hardships	4	(	2	/	0	)	4	(	1	/	0	)
Overloaded with debt	1	(	1	/	0	)	0	(	0	/	0	)
Assumption of excessive debt	0	(	0	/	0	)	0	(	0	/	0	)
Debt (other)	4	(	2	/	0	)	0	(	0	/	0	)
Harassment by debt-collectors	1	(	1	/	0	)	0	(	0	/	0	)
Suicide for death benefit	0	(	0	/	0	)	0	(	0	/	0	)
Other economic-related problems	52	(	44	/	0	)	48	(	44	/	0	)
**Employment problems**	9	(	2	/	0	)	7	(	1	/	0	)
Failure at work	1	(	1	/	0	)	1	(	1	/	0	)
Inter-personal relations at work	2	(	1	/	0	)	3	(	1	/	0	)
Trouble adjusting to changing work environment	0	(	0	/	0	)	1	(	1	/	0	)
Work-related fatigue	0	(	0	/	0	)	0	(	0	/	0	)
Other employment-related problems	6	(	1	/	0	)	2	(	1	/	0	)
**Romantic problems**	206	(	18	/	8	)	137	(	14	/	4	)
Marital problems	0	(	0	/	0	)	0	(	0	/	0	)
Heartbreak	136	(	14	/	4	)	64	(	8	/	1	)
Extra-marital affair	1	(	1	/	0	)	2	(	1	/	0	)
Conflict in relationship	56	(	7	/	0	)	58	(	8	/	0	)
Other romantic-related problems	13	(	3	/	0	)	13	(	4	/	0	)
**School problems**	1089	(	125	/	48	)	493	(	64	/	19	)
Entrance examination problems	139	(	15	/	3	)	52	(	6	/	0	)
Worrying about the future	306	(	29	/	13	)	126	(	20	/	4	)
Underachievement	343	(	43	/	14	)	92	(	15	/	3	)
Inter-personal relations with teachers	22	(	5	/	0	)	14	(	3	/	0	)
Bullying	16	(	3	/	0	)	20	(	4	/	0	)
Conflict with classmate	111	(	16	/	4	)	103	(	19	/	3	)
Other school-related problems	152	(	19	/	6	)	86	(	10	/	2	)
**Others problems**	239	(	26	/	8	)	122	(	15	/	1	)
Public disclosure of crime	23	(	4	/	0	)	9	(	4	/	0	)
Crime victim	1	(	1	/	0	)	4	(	1	/	0	)
Copycat suicide	4	(	1	/	0	)	7	(	2	/	0	)
Loneliness	66	(	7	/	1	)	43	(	5	/	0	)
Neighborhood problems	0	(	0	/	0	)	1	(	1	/	0	)
Others	145	(	17	/	4	)	58	(	8	/	0	)

Supplementary Table s3: Suicide numbers of university students disaggregated by motives and gender during 2007-2022.

	Males			Females		
	Total	(min	/ max)	Total	(min	/ max)
**Total**	5179	397	221	1880	158	86
**Family problems**	378	33	13	157	13	4
Conflict with parent	118	16	3	53	6	1
Marital conflict	5	1	0	4	1	0
Conflict with other family members	43	6	1	30	4	0
Death of family	23	4	0	13	3	0
Hopeless for family	41	6	0	20	3	0
Severe verbal reprimand	83	11	2	19	4	0
Stress of raising children	1	1	0	4	1	0
Physical and/or verbal abuse	1	1	0	0	0	0
Exhaustion from caring for infirm family	2	1	0	0	0	0
Other family-related problems	61	9	1	14	3	0
**Health problems**	1233	123	44	725	60	32
Physical illness	101	13	2	44	4	1
Depression	624	73	18	416	45	15
Schizophrenia	155	19	4	72	8	2
Alcoholism	2	1	0	1	1	0
Drug abuse	3	1	0	4	1	0
Other mental illness	282	25	11	165	17	6
Physical disability	17	4	0	3	1	0
Other health-related problems	49	9	0	20	3	0
**Economic problems**	553	59	19	105	13	1
Bankruptcy	0	0	0	0	0	0
Business struggling	2	1	0	0	0	0
Unemployment	0	0	0	0	0	0
Inability to find employment	345	42	10	75	10	0
Economic hardships	43	6	0	5	1	0
Overloaded with debt	26	4	0	2	1	0
Assumption of excessive debt	0	0	0	0	0	0
Debt (other)	32	6	0	3	2	0
Harassment by debt-collectors	0	0	0	0	0	0
Suicide for death benefit	0	0	0	0	0	0
Other economic-related problems	145	53	2	72	53	0
**Employment problems**	55	8	0	14	3	0
Failure at work	10	2	0	1	1	0
Inter-personal relations at work	11	3	0	5	2	0
Trouble adjusting to changing work environment	5	1	0	0	0	0
Work-related fatigue	5	2	0	6	2	0
Other employment-related problems	24	7	0	2	2	0
**Romantic problems**	325	34	8	196	21	7
Marital problems	12	4	0	4	1	0
Heartbreak	203	20	4	85	8	3
Extra-marital affair	3	2	0	10	3	0
Conflict in relationship	91	12	0	84	8	0
Other romantic-related problems	16	4	0	13	3	0
**School problems**	2193	177	105	541	57	21
Entrance examination problems	35	6	0	6	2	0
Worrying about the future	801	67	33	207	24	6
Underachievement	977	76	43	174	20	3
Inter-personal relations with teachers	26	6	0	8	3	0
Bullying	8	2	0	1	1	0
Conflict with classmate	125	17	4	68	9	1
Other school-related problems	221	22	7	77	9	1
**Others problems**	442	45	17	142	15	2
Public disclosure of crime	35	4	1	1	1	0
Crime victim	2	1	0	0	0	0
Copycat suicide	7	1	0	5	1	0
Loneliness	116	20	2	42	9	0
Neighborhood problems	2	1	0	1	1	0
Others	280	26	12	93	9	2

Supplementary Table s4: Suicide numbers of special vocational school students disaggregated by motives and gender during 2007-2022.

	**Males**						**Females**					
	Total	(	min	/	max	)	Total	(	min	/	max	)
**Total**	1233	(	102	/	58	)	690	(	60	/	27	)
**Family problems**	116	(	12	/	4	)	60	(	7	/	1	)
Conflict with parent	32	(	5	/	0	)	24	(	4	/	0	)
Marital conflict	2	(	1	/	0	)	7	(	2	/	0	)
Conflict with other family members	16	(	3	/	0	)	7	(	2	/	0	)
Death of family	7	(	2	/	0	)	2	(	1	/	0	)
Hopeless for family	12	(	2	/	0	)	4	(	2	/	0	)
Severe verbal reprimand	25	(	4	/	0	)	6	(	3	/	0	)
Stress of raising children	0	(	0	/	0	)	3	(	1	/	0	)
Physical and/or verbal abuse	0	(	0	/	0	)	0	(	0	/	0	)
Exhaustion from caring for infirm family	0	(	0	/	0	)	0	(	0	/	0	)
Other family-related problems	22	(	4	/	0	)	7	(	2	/	0	)
**Health problems**	310	(	29	/	11	)	278	(	30	/	6	)
Physical illness	31	(	4	/	0	)	14	(	2	/	0	)
Depression	152	(	15	/	3	)	151	(	20	/	2	)
Schizophrenia	37	(	5	/	0	)	40	(	6	/	0	)
Alcoholism	1	(	1	/	0	)	1	(	1	/	0	)
Drug abuse	1	(	1	/	0	)	1	(	1	/	0	)
Other mental illness	75	(	9	/	1	)	57	(	11	/	1	)
Physical disability	5	(	1	/	0	)	4	(	1	/	0	)
Other health-related problems	8	(	2	/	0	)	10	(	3	/	0	)
**Economic problems**	130	(	12	/	4	)	25	(	4	/	0	)
Bankruptcy	0	(	0	/	0	)	0	(	0	/	0	)
Business struggling	0	(	0	/	0	)	0	(	0	/	0	)
Unemployment	4	(	2	/	0	)	0	(	0	/	0	)
Inability to find employment	67	(	8	/	1	)	8	(	2	/	0	)
Economic hardships	20	(	3	/	0	)	5	(	2	/	0	)
Overloaded with debt	4	(	1	/	0	)	2	(	1	/	0	)
Assumption of excessive debt	0	(	0	/	0	)	0	(	0	/	0	)
Debt (other)	17	(	3	/	0	)	6	(	2	/	0	)
Harassment by debt-collectors	0	(	0	/	0	)	0	(	0	/	0	)
Suicide for death benefit	0	(	0	/	0	)	0	(	0	/	0	)
Other economic-related problems	60	(	42	/	0	)	46	(	42	/	0	)
**Employment problems**	35	(	4	/	0	)	21	(	4	/	0	)
Failure at work	1	(	1	/	0	)	1	(	1	/	0	)
Inter-personal relations at work	10	(	2	/	0	)	6	(	2	/	0	)
Trouble adjusting to changing work environment	3	(	1	/	0	)	1	(	1	/	0	)
Work-related fatigue	5	(	2	/	0	)	7	(	2	/	0	)
Other employment-related problems	16	(	2	/	0	)	6	(	3	/	0	)
**Romantic problems**	110	(	10	/	3	)	95	(	14	/	3	)
Marital problems	1	(	1	/	0	)	2	(	1	/	0	)
Heartbreak	74	(	8	/	2	)	48	(	8	/	0	)
Extra-marital affair	2	(	1	/	0	)	6	(	1	/	0	)
Conflict in relationship	28	(	5	/	0	)	31	(	3	/	0	)
Other romantic-related problems	5	(	2	/	0	)	8	(	2	/	0	)
**School problems**	434	(	40	/	18	)	175	(	18	/	6	)
Entrance examination problems	57	(	8	/	1	)	10	(	2	/	0	)
Worrying about the future	153	(	17	/	3	)	41	(	5	/	0	)
Underachievement	155	(	16	/	5	)	61	(	8	/	1	)
Inter-personal relations with teachers	4	(	2	/	0	)	7	(	1	/	0	)
Bullying	3	(	1	/	0	)	5	(	1	/	0	)
Conflict with classmate	25	(	5	/	0	)	33	(	6	/	0	)
Other school-related problems	37	(	5	/	0	)	18	(	5	/	0	)
**Others problems**	98	(	11	/	3	)	36	(	6	/	0	)
Public disclosure of crime	6	(	2	/	0	)	0	(	0	/	0	)
Crime victim	1	(	1	/	0	)	0	(	0	/	0	)
Copycat suicide	3	(	1	/	0	)	2	(	1	/	0	)
Loneliness	22	(	4	/	0	)	18	(	4	/	0	)
Neighborhood problems	0	(	0	/	0	)	0	(	0	/	0	)
Others	66	(	7	/	2	)	16	(	3	/	0	)

Supplementary Fig.s1: SMRs caused by suicidal motives of high school, university and special vocational school students of males and females during 2007–2022.

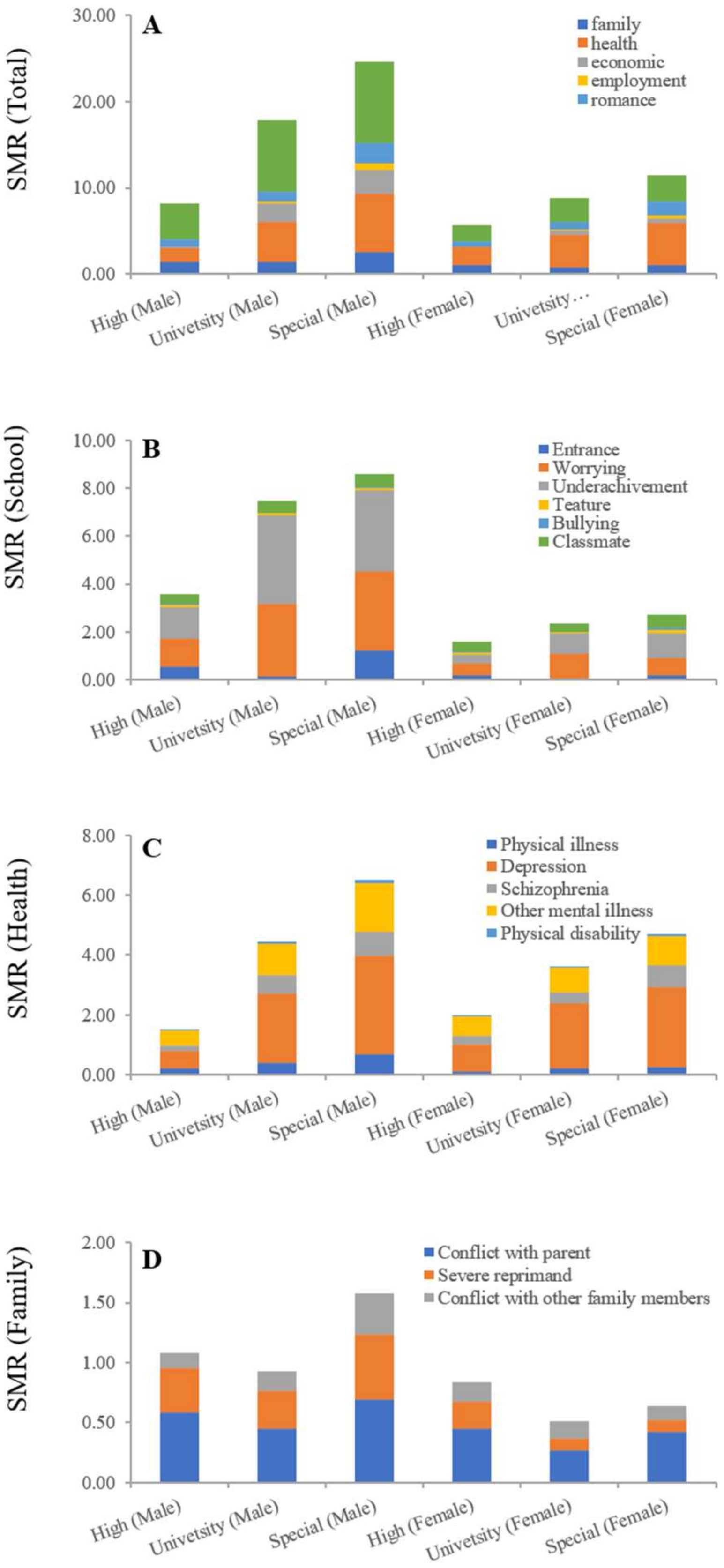

Average of SMRs of high school, university and special vocational school students of males and females during 2007–2022 caused by major 6 suicidal motives (A), subcategories in school-related motives (B), health-related motives (C) and family-related motives (D) were represented. The ordinates indicate the mean of SMRs (per 100,000 population) during 2007-2022.

Supplementary Fig.s2: Trends of SMRs caused by 6 major suicidal motives during 2007-2022.

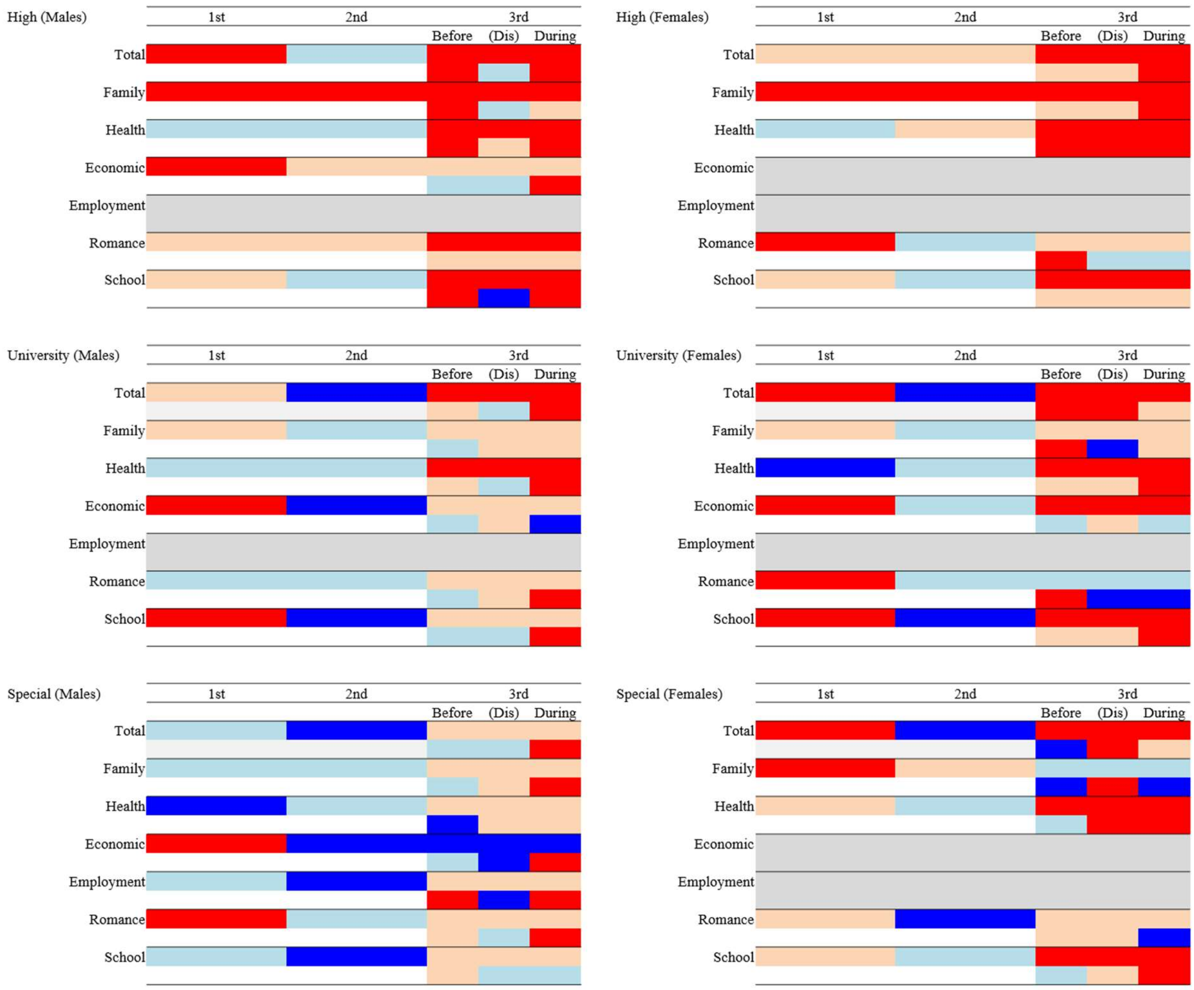

Trends of SMRs of high school (High), university and special vocational school (Special) students of males and females disaggregated by 6 major motives (total, family-, health-, economic-, employment-, romance- and school-related motives) during 1st (2007-2012), 2nd (2012-2017) and 3rd GPSPP (2017-2022) were indicated in upper columns. Especially, to analyse the impacts of the COVID-19 pandemic, trends and discontinuity (Dis) of SMRs between before (2017-2020) and after (2020-2022) the pandemic outbreak were lower columns. Blue and red columns indicate significant ($p < 0.05$) decreasing and increasing trends of SMR detected by ITSA. Light blue and red columns indicate significant decreasing and increasing trends detected by ITSA. Grey columns indicated the excluded categories. Actual trends of SMRs detected by ITSA were indicated in Supplementary Fig.2.

Supplementary Fig.s3: Trends of SMRs caused by suicidal motives in school-, health- and family-related problems during 2007-2022.

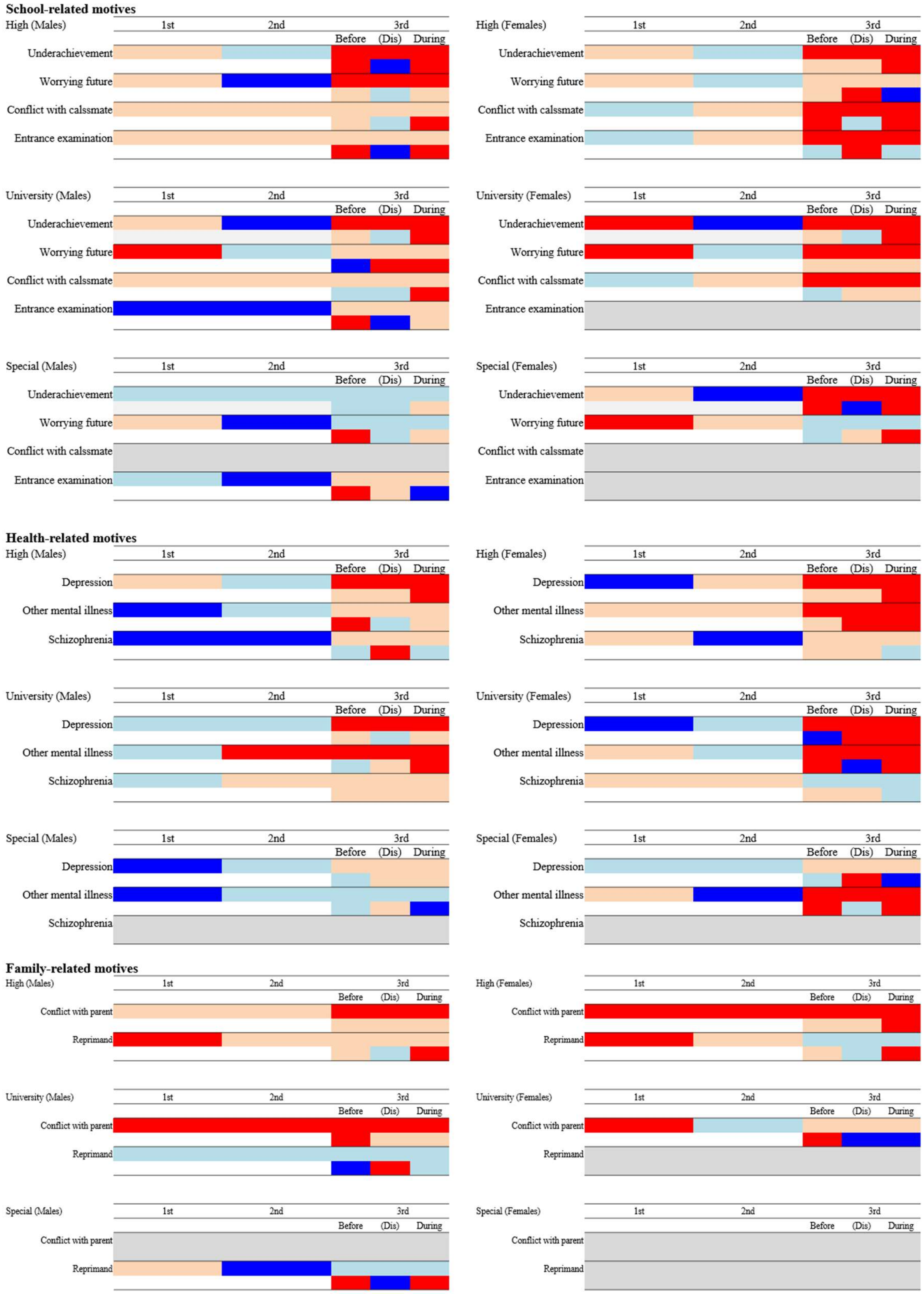

Trends of SMRs of high school (High), university and special vocational school (Special) students of males and females disaggregated by suicidal motives (in family-, health- and school-related motives) during 1st (2007-2012), 2nd (2012-2017) and 3rd GPSPP (2017-2022) were indicated in upper columns. Especially, to analyse the impacts of the COVID-19 pandemic, trends and discontinuity (Dis) of SMRs between before (2017-2020) and after (2020-2022) the pandemic outbreak were lower columns. Blue and red columns indicate significant ($p < 0.05$) decreasing and increasing trends of SMR detected by ITSA. Light blue and red columns indicate significant decreasing and increasing trends detected by ITSA. Grey columns indicated the excluded categories. Actual trends of SMRs caused school-, health- and family-related motives by detected by ITSA were indicated in Figs.3-4.

Table: Effects of grade repetitions on SMRs of university students caused by suicidal motives

		Male						Female					
Factor	(Subcategory)	Adjusted R^2	F value	P value	Year	β	P value	Adjusted R^2	F value	P value	Year	β	P value
Total		0.541	12.760	0.001 **	R1	-1.91	0.72	0.710	5.268	0.015 *	R1	-17.31	0.580
					R2	43.14	0.11				R2	90.08	0.309
					R3	3.08	0.92				R3	-185.08	0.400
					R4<	-125.83	0.11				R4<	-207.76	0.269
Family		-0.177	1.308	0.331	R1	0.67	0.518	0.304	3.484	0.050 *	R1	-2.02	0.101
					R2	-2.51	0.493				R2	15.37	0.005 **
					R3	-1.58	0.862				R3	-32.30	0.051
					R4<	14.71	0.174				R4<	23.07	0.122
	Conflict with parent	-0.029	1.633	0.248	R1	-0.584	0.228	-0.156	1.793	0.207	R1	0.037	0.981
					R2	2.806	0.094				R2	2.178	0.631
					R3	-4.293	0.293				R3	-16.280	0.280
					R4<	-0.993	0.791				R4<	-0.406	0.947
	Reprimand	-0.273	0.785	0.560	R1	0.358	0.234	-0.292	2.647	0.097	R1	-0.283	0.057
					R2	-1.819	0.233				R2	3.278	0.072
					R3	0.992	0.661				R3	-4.774	0.576
					R4<	4.157	0.410				R4<	6.180	0.511
Health		0.243	3.612	0.045 *	R1	-5.587	0.010 *	0.603	25.779	0.000 **	R1	2.83	0.716
					R2	19.832	0.048 *				R2	-24.66	0.240
					R3	32.011	0.062				R3	-51.85	0.499
					R4<	-111.059	0.009 **				R4<	-199.39	0.005 **
	physical	0.690	7.433	0.005 **	R1	-1.34	0.000 **						
					R2	1.00	0.559						
					R3	10.42	0.084						
					R4<	-10.67	0.111						
	depression	0.316	3.774	0.040 *	R1	-4.166	0.063	0.506	7.929	0.004 **	R1	-1.30	0.873
					R2	20.405	0.043 *				R2	-19.34	0.451
					R3	11.411	0.651				R3	-3.25	0.961
					R4<	-79.193	0.036 *				R4<	-170.59	0.016 *
	schizophrenia	0.101	3.946	0.036 *	R1	-0.41	0.272	-0.037	3.532	0.048 *	R1	3.03	0.020 *
					R2	2.21	0.258				R2	-4.34	0.337
					R3	4.95	0.234				R3	-29.28	0.104
					R4<	-18.97	0.036 *				R4<	-16.57	0.205
	Other mental illness	-0.114	1.402	0.302	R1	0.370	0.451	-0.351	0.750	0.581	R1	-0.282	0.929
					R2	-2.171	0.338				R2	-2.312	0.782
					R3	2.424	0.681				R3	3.266	0.913
					R4<	-3.441	0.574				R4<	-5.642	0.795
Economic		0.091	2.567	0.103	R1	2.74	0.278	0.035	0.915	0.492	R1	0.27	0.950
					R2	3.17	0.732				R2	11.09	0.508
					R3	-16.75	0.198				R3	-45.59	0.096
					R4<	-1.61	0.930				R4<	1.43	0.957
Employment		0.136	5.669	0.012 *	R1	0.12	0.726	-0.303	0.433	0.782	R1	1.27	0.329
					R2	1.42	0.291				R2	-1.52	0.665
					R3	-1.00	0.816				R3	-10.84	0.356
					R4<	-2.56	0.516				R4<	-5.33	0.447
Romance		0.177	1.438	0.291	R1	-0.69	0.757	0.162	1.399	0.303	R1	3.40	0.324
					R2	2.95	0.738				R2	3.61	0.773
					R3	9.93	0.489				R3	-74.40	0.061
					R4<	-27.71	0.164				R4<	-17.20	0.589
	conflict with partner	-0.065	1.311	0.330	R1	-0.506	0.164	0.816	81.958	0.000 **	R1	2.24	0.000 **
					R2	-0.817	0.604				R2	2.86	0.255
					R3	7.482	0.084				R3	-44.70	0.000 **
					R4<	-6.643	0.118				R4<	-7.60	0.205
School		0.225	11.652	0.001 **	R1	-4.78	0.203	0.274	3.569	0.047 *	R1	-8.12	0.066
					R2	16.37	0.140				R2	43.54	0.025 *
					R3	32.09	0.049 *				R3	-41.82	0.456
					R4<	-66.15	0.108				R4<	-34.17	0.605
	worrying about the future	0.660	16.295	0.000 **	R1	2.80	0.007 **	0.131	3.935	0.036 *	R1	-3.56	0.011 *
					R2	0.35	0.933				R2	4.24	0.594
					R3	-5.23	0.551				R3	46.30	0.036 *
					R4<	-5.65	0.561				R4<	-19.41	0.469
	underachievement	0.387	6.851	0.006 **	R1	-3.39	0.058	0.171	3.789	0.040 *	R1	-2.46	0.576
					R2	13.37	0.047 *				R2	29.29	0.049 *
					R3	7.90	0.514				R3	-75.21	0.060
					R4<	-30.03	0.059				R4<	13.01	0.755
	Conflict with classmate	0.481	2.859	0.081	R1	-0.251	0.811	-0.049	3.629	0.045 *	R1	0.747	0.783
					R2	-0.417	0.909				R2	9.058	0.273
					R3	2.701	0.452				R3	-25.506	0.325
					R4<	5.354	0.329				R4<	-12.362	.0377

*: $p<0.05$, **: $p<0.01$: analysed by multiple regression analysis with robust standard error.

R1: repeat the same grad for one year, R2: repeat the same grad for two year, R3: repeat the same grad for three year, R4<: repeat the same grad for more than four years.
